# Monthly Summary

**Published:** 1870-07

**Authors:** 


### MONTHLY SUMMARY.

*Bromide of Potassium for the Troubles of Teething.*—The bromide of potassium is highly spoken of by Dr. Hoehling (*St. Louis Med. Jour.*) and by Dr. Caro (*Medical Record*), as a remedy for faulty nutrition, irritability of the stomach, vomiting, restlessness, diarrhœa, etc., resulting from teething. From half a grain to a grain, given every hour or two, will, in a short time often procure relief and sleep when other means have failed. The following is selected from Dr. Caro's list of twenty cases: S. Fay nine months old, has six teeth, nursed by a healthy mother. After fifteen days of diarrhœa with from fifteen to twenty passages every twenty-four hours, was cured by a mixture of one drachm of bromide of potassium in an ounce of mucilage, twenty drops every hour. Dr. Caro adds: "I have had fifteen similar cases in every form, having been sick from fifteen to twenty days before coming to me, all treated and cured by the bromide of potassium."—*Chicago Medical Journal.*

---

*Cystic Tumor of the Lower Jaw.*--Dr. H. F. Lyster, of Detroit by request, exhibited to the Michigan State Dental Association a cystic tumor, composed of a portion of the lower jaw which he successfully removed in April last, in the interior of the State. From the left canine to the third molar the jaw had been expanded, on every side, to the size and thickness of a hen's egg. This change had been going on for seven years, and was supposed to have originated in irritation from an uncut tooth; and the fluid of the tumor had been evacuated several times for the relief of pain. Upon opening the tumor, after removal, a foreign body, about the size of a molar tooth, was discovered; which proved, upon examination, to be composed of salivary calculus. The operation of removal consisted in making an incision along the lower border of the tumor, and dissecting off the soft parts with the periosteum, and using a metacarpal saw and bone forceps just anterior to the canine and posteror to the third molar. The opening into the cavity of the mouth was made as late as possible to avoid hemorrhage into the pharynx. By keeping closely to the tumor, hemorrhage was avoided, and no vessel required a ligature. The dental artery was plugged in its bony canal. The dental nerve was discovered stretched across the interior of the tumor in its normal condition, but unsupported by any tissues. The tumor was filled with a mucous fluid of the appearance of saliva. The position of the chin and the contour of the face had been well preserved by moulding on binders board, and by wiring the teeth on the sound side.—*Dental Register.*

*Bromide of Potassium in Dentition.*—Dr. Salvatore Caro, in the *Medical Record*, strongly recommends the local application of this remedy to allay irritation of the gums in dentition. He says: "In the most severe cases of odontitis, either with or without ulcerated gums and loose bowels, I have never failed to relieve the child by the application of the bromide of potassium. Almost immediately after the first rubbing of the gums, from being turgid, swollen and red, they assume their natural color, and a certain amount of ease is felt. Saliva commences to dribble; and, as if by enchantment, agitation, carpopedal involuntary motion, vomiting and loosensss of the bowels disappear. As the vomiting and diarrhœa in this case are not the consequence of gastro-enteritis, but of an excitement of the stomach and the intestinal mucous membrane, owing to the inflamed condition of the gums, I suppose it will never be cured, either by scarification of the gums, or by the use of astringents or anodynes; but, as I shall hereafter prove, simply by the use of bromide of potassium."

---

*Stomatitis Produced by Fermented Cheese, and Cured by Lemon Juice.*—A store servant, having good antecedents, *i. e.*, having never had recourse to mercurial preparations, was brought in the service of Behier, on account of violent stomatitis. In examining the mouth, no aphthæ nor ulcerations, or grayish patches, nothing of a pultaceous nature. What was most striking was a universal brilliant redness with loss of epithelium, without tumefaction of the tissues, covered by the mucous membranes. This was alone affected. Finally, this was a simple erythematic stomatitis, but of exceptional intensity.

The patient attributed, and with reason, this buccal lesion to an excess in the eating of fermented cheese In fact, we often meet with dried and fermented cheese, particularly those of Rocquefort and Gruyere which acquire with age irritant properties similar to those of musta[illegible]d, and produce a stomatitic affection of the nature of that of which we treat.

However, happily, this is more painful than grave. It is worthy of remark, however, that when cured, the patients preserve an extreme susceptibility of the buccal mucous membranes, especially to salted ailments.

How shall we treat the stomatitis? by substitution. If we prescribe emollients, decoction of figs, marshmallow or poppy, etc., the sanguineous tufts persist with tenacity. We should them make use of excitants, alum, chlorate of potash, and beyond all citrons. In the patient of Behier the mouth was energetically touched in every direction with a piece of lemon. The blood flowed from the papillæ, but a notable change for the better was immediate, and the cure was complete on the following day.—*Journal de Medicine et Chirurgie*' and *New Orleans Jour. Medicine.*

---

*Ulceration of the Palate in Young Patients.*—In November last, a stout healthy looking girl, eleven years of age, was under treatment in the surgical wards of the London Hospital, for ulceration at the back of the mouth, which resulted in destruction of the uvula and the adjacent soft parts. The Professor stated to his class that as ulceration and destruction of the soft palate in an adult generally indicates tertiary syphilis, so does the existence of a similar disease in a young subject at once suggest the idea of hereditary syphilitic taint. But it has been observed that destruction of the palate at an early age is very rarely if ever associated with the signs of inherited syphilis. In the present patient the teeth were not deformed, there was no keratitus, and periosteal thickenings could not be detected. If, as we have good reasons for believing, early ulceration be due to hereditary syphilis, then this lesion characterizes a group of cases quite distinct from that in which the ordinary symptoms of inherited disease, such as pegged teeth and keratitus, are presented. The most effective treatment of slonghing of the soft palate in young patients is the local application of nitric acid or the acid nitrate of mercury.—*Lancet.*

---

*Death from Chloroform.*"—At Accrington, on Saturday week, a young woman aged 30, named Susanna Horsfall, went to Dr. Millar's surgery to have some teeth extracted. The teeth were difficult to extract, and chloroform was administered. After having pulled out the third tooth, Dr. Millar observed that the patient was dying; and life was soon extinct. She had taken chloroform a week before, but it did not take effect."—*British Medical Journal.*

*Facial Neuralgia.*—While practicing medicine at Leiganore, Maryland, I was called 45 miles over into the vicinity of Gettysburg, Penna., sometime in April 1863 to see Mrs. Rachel Pfoutz, a lady of excellent standing and wide circle of aquaintance.

The history of this case is, that for several years previous she had been slightly affected with neuralgia, which about six months before I was called in had concentrated its forces in the dental and lingual nerves.

The skill of five physicians of good standing had failed to relieve her, and she had been abandoned as a hopeless case. Now, she could neither speak nor masticate for over two months, conversed solely by slate and pencil—while her subsistence was effected by sucking through a glass tube, a portion of wine soup and other fluid diet. Death she said was her hope. A person weighing in health 218 pounds, was now reduced to 80 pounds. Her treatment, which had been of the anti-periodic, and anti-spasmodic, and narcotic kind, had over-ruled the peristaltic action of the bowels, and the evacuations depended solely on injections. So sensitive was her nervous system that the least sudden noise produced spasms.

I saw clearly what was at first to be done—accordingly prescribed pil. comp. cath. ij., followed by sal. Rochelle ℥j. The bowels soon resumed their wonted duty, and now for a removal of the intense pain, and a tonic for the general system.

These I found in tinct. lupulin, ℥ij, teaspoonful in water every 5 hours—alternating with syr. iodid. ferri, ℥j, 10 drops in water, and aconitin, gr. j, hog's lard, ʒj. M. Ft. ung. Applied over the seat of pain. She spoke instantly, and said she was cured. The pain ceased instantly upon the first application of the ointment.

Her improvement under this course was rapid, and soon she was well. But I found in June following that there was a disposition in the nerves to resume the painful task, as the aconitin began to lose its effect.

I determined to operate at once, and addressed to Prof. S. D. Gross, of the Jefferson Medical College, a letter as to the matter. On receiving his reply, I took my patient to Philadelphia. The eminent Professor named, incised, trephined, divided behind the bifurcation, and lifted out the dental nerve.

The patient returned home in a week, enjoyed good health a long time, but from a letter I received from her a few years ago, she tells me she suffers again some at times, but no comparison to the first—as life is since a pleaure, whereas, before our treatment, 'twas but a living death. I believe such treatment worthy of adoption.—*Dr. P. J. Gardiner in Medical & Surgical Reporter.*

---

*The Oldest Relic of Humanity.*—The oldest relic of humanity extant is the skeleton of one of the earlier Pharaohs, encased in its original burial robes, and wonderfully perfect considering its age, which was deposited about 18 or 20 months ago in the British Museum, and is justly considered the most valuable of its archæological treasures. The lid of the coffin which contained the royal mummy was inscribed with the name of its occupant, Pharaoh Mikerinus, who succeeded the heir of the builder of the Great Pyramid, about ten centuries before Christ. The monarch whose crumbling bones and leathery integuments are now exciting the wonder gazers in London, reigned in Egypt before Solomon was born, and only about 11 centuries or so after Mizraim, the grandson of father Noah, and the first of the Pharaohs, had been gathered to his fathers The tidemark of the deluge could scarcely have been obliterated when this man of the early world lived, moved and had his being.—*Medical Record.*

---

*Sulphate of Nickel in Neuralgia.*—A case of obstinate neuralgia is related which was cured by sulphate of Nickel, in doses of half a grain, three times a day. At the end of a week a dose of one grain was given. Its sedative action was speedily manifested in reducing the pulse and procuring sleep. All symptoms of the paroxysm disappeared. The remedy seems worth a trial.—*Physician and Pharmaceutist.*

---

